# Nectary size is a pollination syndrome trait in *Penstemon*

**DOI:** 10.1111/nph.15769

**Published:** 2019-03-26

**Authors:** Amanda M. Katzer, Carolyn A. Wessinger, Lena C. Hileman

**Affiliations:** ^1^ Department of Ecology and Evolutionary Biology University of Kansas 1200 Sunnyside Avenue Lawrence KS 66045 USA

**Keywords:** complex phenotype, evolution, nectar volume, nectary development, *Penstemon*, phenotypic correlation, pollination syndrome

## Abstract

- Evolution of complex phenotypes depends on the adaptive importance of individual traits, and the developmental changes required to modify traits. Floral syndromes are complex adaptations to pollinators that include color, nectar, and shape variation. Hummingbird‐adapted flowers have evolved a remarkable number of times from bee‐adapted ancestors in *Penstemon*, and previous work demonstrates that color over shape better distinguishes bee from hummingbird syndromes. Here, we examined the relative importance of nectar volume and nectary development in defining *Penstemon* pollination syndromes.
- We tested the evolutionary association of nectar volume and nectary area with pollination syndrome across 19 *Penstemon* species. In selected species, we assessed cellular‐level processes shaping nectary size. Within a segregating population from an intersyndrome cross, we assessed trait correlations between nectar volume, nectary area, and the size of stamens on which nectaries develop.
- Nectar volume and nectary area displayed an evolutionary association with pollination syndrome. These traits were correlated within a genetic cross, suggesting a mechanistic link. Nectary area evolution involves parallel processes of cell expansion and proliferation.
- Our results demonstrate that changes to nectary patterning are an important contributor to pollination syndrome diversity and provide further evidence that repeated origins of hummingbird adaptation involve parallel developmental processes in *Penstemon*.

## Introduction

Pollination syndromes are suites of floral traits that attract, reward, and facilitate pollination by a particular type of animal or abiotic agent (Faegri & van der Pijl, 1979; Fenster *et al*., 2004). Elements of a pollination syndrome include floral pigmentation, nectar offerings, scent production, and flower shape. For example, moth‐pollinated flowers have a sweet fragrance, reflective white corollas, and a narrow tubular shape. Evolutionary transitions from one syndrome to another therefore require changes to not just one, but to multiple floral traits. Despite this complexity, many plant genera show repeated transitions in pollination syndrome (Thomson & Wilson, 2008; Abrahamczyk & Renner, 2015). Studies that address floral evolution associated with pollinator adaptation provide key insights into the relative importance of individual floral traits, and a deeper understanding of the genetic and developmental changes required for syndrome evolution.

A common pollination‐syndrome shift in the North American flora is from bee adaptation to hummingbird adaptation (Thomson & Wilson, 2008). These transitions appear to occur over short evolutionary timescales (Beardsley *et al*., 2003; Whittall & Hodges, 2007; Wilson *et al*., 2007; Gübitz *et al*., 2009; Soza *et al*., 2012), and several North American genera include sister species that display bee vs hummingbird syndrome (Abrahamczyk & Renner, 2015). Shifts from bee to hummingbird syndrome have been extensively studied in the North American genus *Penstemon*, in which species largely conform to either bee syndrome (the ancestral condition) or hummingbird syndrome (Wilson *et al*., 2004). Bee‐adapted *Penstemon* flowers are generally blue or purple and produce small amounts of nectar. Their floral tubes are relatively wide, and the flowers are positioned horizontally with the lower petal lobe forming a landing platform. The stamen filaments and style are relatively short, such that the anthers and stigma are located within the flower. Hummingbird‐adapted *Penstemon* flowers are bright red to magenta and produce large amounts of nectar. The floral tubes are narrow, and the flowers are inclined downwards with the lower petal lobes reduced or reflexed, eliminating an obvious landing platform. The stamen filaments and style are elongated so that the anthers and stigma are exserted outside the corolla tube. Transitions from bee to hummingbird syndrome have occurred an estimated 15–20 times during *Penstemon* diversification (Wilson *et al*., 2007).

Given the remarkably large number of pollination‐syndrome transitions, *Penstemon* is a useful system for understanding the contribution of individual traits to pollination‐syndrome evolution. Wilson *et al*. (2004) amassed floral phenotypic data and pollinator visitation for a broad sample of *Penstemon* species and found that flower color is a primary predictor of pollinator visitation, much more so than floral dimension traits such as corolla width and corolla length. This is consistent with the substantial variation in flower size and shape, but not flower color, within each pollination syndrome (Wilson *et al*., 2004). Flower color functions as a signal for both bee and hummingbird visitors (reviewed by Wilson *et al*., 2006; Wilson & Jordan, 2009), and previous studies have investigated the genetic and developmental basis of transitions from blue–purple to red *Penstemon* flowers. These transitions involve loss‐of‐function mutations to the anthocyanin pathway enzyme flavonoid 3′,5′‐hydroxylase that is responsible for converting precursors of red pigments into precursors of blue pigments (Wessinger & Rausher, 2014, 2015). Loss‐of‐function mutations are expected to have large mutational target sizes, which helps explain how transitions in flower color have repeatedly occurred over short evolutionary timescales.

Changes in flower color are, however, not sufficient to drive shifts to hummingbird pollination. Importantly, hummingbird visitation depends on nectar offerings. Hummingbirds directly respond to the volume of nectar, and this response can override signals provided by flower color (Schemske & Bradshaw, 1999; Wilson & Jordan, 2009). This observation has led to the hypothesis that transitions to hummingbird pollination are initiated by changes in nectar volume, followed by changes to color as a reinforcing signal, followed by later changes to flower shape to improve pollen transfer efficiency (Wilson *et al*., 2006; Thomson & Wilson, 2008). Under this hypothesis, increased nectar volume should be strongly associated with hummingbird syndrome in *Penstemon*. Here, we build on a preliminary qualitative comparison (Thomson *et al*., 2000) by quantifying the association between nectar production and pollination syndrome across *Penstemon*.

Compared with our understanding of *Penstemon* flower color evolution, little is known about the genetic and developmental basis of variation in *Penstemon* nectar production. In a *Penstemon* flower, nectar is produced by two patches of glandular trichomes located on the bases of the lateral stamen filaments (Straw, 1966) (Supporting information Fig. S1), and can be replenished within 2–3 h after depletion by a visiting pollinator (Castellanos *et al*., 2002). This dynamic nectar production may be an important trait for adaptation to pollinators with high energetic demands, such as hummingbirds. However, it is unknown whether changes to nectary morphology or physiological processes of nectar secretion, or both, underlie adaptive change in nectar volume. Our previous quantitative trait locus (QTL) mapping study in a segregating F_2_ population from a cross between *Penstemon neomexicanus* (bee syndrome) and *Penstemon barbatus* (hummingbird syndrome) found that nectar volume variation has a simple genetic basis (Wessinger *et al*., 2014). This suggests that increased nectar production can be accomplished through relatively few genetic changes. However, potential developmental correlates of variation in nectar volume have not been explored. We hypothesize that transitions to hummingbird pollination in *Penstemon* may be enabled by a nectary structure and function that can easily be modified to increase nectar offerings. Under this hypothesis, we expect to see similar changes to nectary morphology and/or physiology across independent origins of hummingbird adaptation in *Penstemon*.

**Figure 1:**
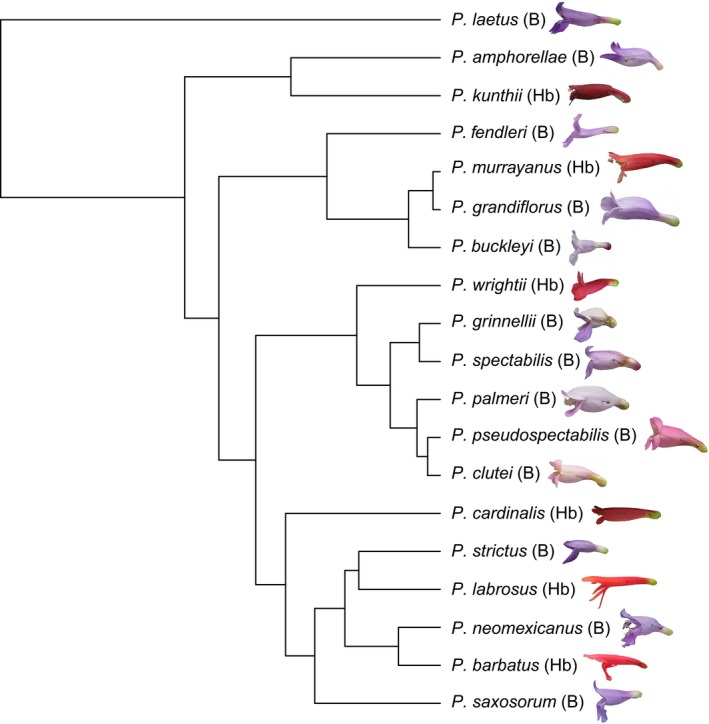
Relationships among 19 sampled *Penstemon* species and their corresponding pollination‐syndrome designations: B, bee‐pollination syndrome; Hb, hummingbird‐pollination syndrome.

Here, we test for an association of nectar volume with pollination syndrome in *Penstemon* using a phylogenetic comparative framework. Across 19 species that include six independent origins of hummingbird syndrome (Fig. 1), we find that nectar volume is strongly associated with pollination syndrome. In addition, we begin to identify developmental correlates of increased nectar production by focusing first here on developmental patterning of the nectary itself. We find that the area of the glandular trichome nectary predictably accompanies the evolution of larger nectar volume across *Penstemon*. We show that increases in glandular trichome size and trichome proliferation contribute to overall increases in nectary area. Using an F_2_ mapping population, we find that nectary area is positively correlated with nectar volume, demonstrating that increases in nectary area resulting from cell expansion and proliferation processes likely facilitate evolutionary shifts in nectar production critical for hummingbird attraction. We also find that nectary area and nectar volume are positively correlated with lateral stamen length. This architecture may ease transitions to hummingbird adaptation, since the trait correlation is parallel to the direction of natural selection.

## Materials and Methods

### Species sampling and assignment of pollination syndrome

We sampled 19 *Penstemon* species representing six independent transitions to hummingbird syndrome (Fig. 1). Previous studies clearly demonstrated that when *Penstemon* species are plotted in multidimensional trait space they separate into two distinct clusters that correspond to bee vs hummingbird syndrome with little ambiguity (Wilson *et al*., 2004). Therefore, we assigned our sampled species to one of these two pollination syndromes according to the designations provided by Wilson *et al*. (2004, 2007) (Fig. 1). Two species in our dataset exhibit floral traits consistent with intermediate pollination syndrome, *Penstemon clutei* and *Penstemon pseudospectabilis*. Following the strict definition of bee vs hummingbird syndrome (Wilson *et al*., 2007), we treated these species as bee syndrome.

### Phylogeny

We previously inferred a phylogeny for a set of 124 *Penstemon* species on a concatenated alignment of multiplexed shotgun genotyping data (Wessinger *et al*., 2016) (C. A. Wessinger *et al*., unpublished). We pruned this to include just the 19 sampled species and rescaled branch lengths to be proportional to time (ultrametric) using the chronos() function in the R package ape (Paradis *et al*., 2004). The alignment and tree file have been deposited in Dryad, doi: 10.5061/dryad.99s9r6b.

### Flower phenotypic measurements

We measured a suite of floral traits commonly associated with *Penstemon* pollination syndrome. We scored flower color on a scale of 1–4 following Wilson *et al*. (2004) (Table S1). Briefly, 1 = blue‐purple, 2 = light purple or pink, 3 = magenta, and 4 = red. We performed quantitative floral trait measurements on at least three flowers per individual plant (one to three individuals per species). All plants were grown to flowering under glasshouse conditions with supplemental lighting (14 h day length.) We recorded all floral measurements on the morning of anthesis, between 09:00 h and 12:00 h. We measured nectar volume using 5 μl microcapillary tubes. We measured floral tube length as the distance from the base of the corolla to the lateral sinus connecting lateral to dorsal petal lobes, and floral tube width as the lateral distance across the corolla tube opening. Measurements were taken by first digitally photographing floral organs with a ruler for calibration and then corolla tube length and width dimensions were measured using fiji software (Schindelin *et al*., 2012). We stored lateral stamen filaments in 70% ethanol up to 1 month and photographed nectaries (at the base of lateral stamens) using a Lumenera Infinity 3s camera attached to a Leica MZ16F dissecting scope, with a slide micrometer for calibration. We later measured nectary area in fiji. For some plants we measured nectar volume from more than three flowers if measurements were highly variable. All quantitative trait measurements were log‐transformed for downstream analyses. Sample size, mean, and variance for each trait across sampled species are listed in Table S1.

### Evolutionary associations between floral traits and pollination syndrome

We used phylogenetic ANOVA and phylogenetic linear regression (LR) to test for associations between continuous floral traits and pollination syndrome using the phylANOVA() function in phytools (Revell, 2012) and the phylolm() function in the R package phylolm (Ho & Ane, 2014), respectively. Pollination syndrome was treated as a binary trait with bee syndrome scored as state 0 and hummingbird syndrome as state 1. The phylogenetic ANOVA uses Brownian motion to model phylogenetic signal. The LR approach allows us to examine additional evolutionary models. For each association tested by LR we fit the following evolutionary models: (1) Pagel's lambda (PL), where the rate of trait evolution is optimized from the data; (2) Brownian motion, where traits evolve according to random drift; and (3) Ornstein–Uhlenbeck, where traits evolve towards an optimum. We compared model fit using the Akaike information criterion and used the model with the best fit to estimate LR associations.

We used phylogenetic generalized least squares (PGLS) to test for an association between nectar volume and nectary area using the gls() function in R package nlme (Pinheiro *et al*., 2015). To account for phylogenetic structure, we fit the same PGLS trait evolution models described earlier. We compared model fit using the Akaike information criterion and used the model with the best fit to estimate the association between nectar volume and nectary area.

### Nectary cell size measurements

We compared the size of nectary glandular trichome cells from two species pairs that represent independent origins of hummingbird syndrome. These two species pairs are (1) *P. neomexicanus* (bee syndrome) and closely related *P. barbatus* (hummingbird syndrome) and (2) *Penstemon amphorellae* (bee syndrome) and closely related *Penstemon kunthii* (hummingbird syndrome). We fixed lateral stamens of three flowers from one to three plants of each species in FAA (50% ethanol, 10% formaldehyde (37%), 5% glacial acetic acid). For our analysis, we collected data from four to six nectaries per species. We dehydrated through an ethanol series and embedded fixed stamens in paraffin wax for sectioning. Four 10 µm transverse sections representing the top, middle, and bottom of each nectary (Fig. S1) were mounted on microscope slides. Sections were de‐waxed in Citrisolve (Thermo Fisher Scientific, Pittsburgh, PA, USA) and rehydrated through an ethanol series. For each tissue section, we quantified the width of four segments, each five cells across, for a total of 16 five‐cell segments per nectary (Fig. S1). This approach is similar to petal cell size measurements in Ding *et al*. (2017). Measurements were recorded directly on infinity analyze software using a Zeiss Axioskop 2 plus microscope with a Lumernera Infinity 3 camera. We tested for differences in nectary trichome cell size between the bee‐ and hummingbird‐adapted member of each species pair using Welch's two‐sample *t*‐test with the function t.test() in R (R Core Team, 2013).

### Trait correlations in the *P. amphorellae* × *P. kunthii* F_2_ population

We crossed *P. amphorellae* to *P. kunthii* and self‐pollinated the F_1_ progeny to generate a population of F_2_ individuals. We measured nectar volume, nectary area, and lateral stamen filament length on three flowers for each of 46 F_2_ individuals. We determined nectar volume and nectary area as described earlier. We determined filament length by digitally photographing stamens with a ruler for calibration and measuring filament length using fiji. We log‐transformed all measurements, averaged trait values across flowers for each individual, and then calculated pairwise correlations between nectar volume, nectary area, and stamen filament length with the cor.test() function in R (R Core Team, 2013).

## Results

### Evolutionary associations between floral traits and pollination syndrome

**Figure 2:**
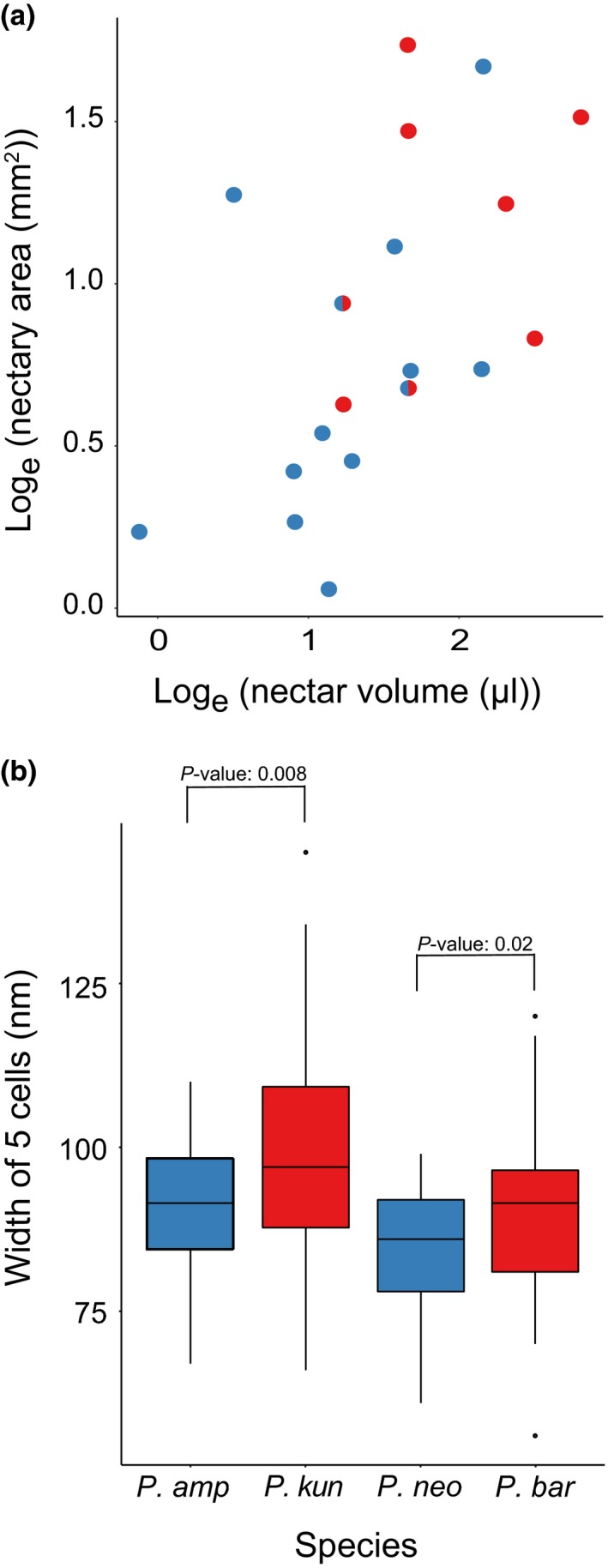
Evolutionary associations for nectar traits. (a) Scatterplot depicting the association between nectar volume and nectary area across 19 *Penstemon* species. Bee‐syndrome species with blue circles, hummingbird‐syndrome species with red circles, and *Penstemon clutei* and *Penstemon pseudospectabilis* are depicted by a circle that is half red and half blue since they have flowers consistent with intermediate syndrome, but they were treated as bee syndrome according to Wilson *et al*. (2007) in our evolutionary association analyses. (b) Nectary trichome cell width, measured in five cell units, for two species pairs with contrasting pollination syndromes. Box plots depict median values (horizontal lines), first and third quartiles (upper and lower hinges), range of values not exceeding 1.5× the interquartile range (upper and lower whiskers), and outliers (upper and lower dots). Bee‐syndrome species are shown in blue; hummingbird‐syndrome species are shown in red. *P. amp*,* Penstemon amphorellae* (*n* = 4 nectaries, two flowers); *P. kun*,* Penstemon kunthii* (*n* = 6 nectaries, three flowers); *P. neo* = *Penstemon neomexicanus* (*n* = 4 nectaries, two flowers); *P. bar*,* Penstemon barbatus* (*n* = 5 nectaries, three flowers).

We found that nectar volume and nectary area are each significantly associated with pollination syndrome according to both phylogenetic ANOVA and LR analyses (Table 1). This provides clear evidence that nectar traits are a component of the pollination syndrome. In addition, we recovered the previously identified strong association between flower color and pollination syndrome (Table 1) (Wilson *et al*., 2004). Wilson *et al*. (2004) found that corolla tube length, but not corolla tube width, was moderately associated with pollination syndrome. Our phylogenetic ANOVA and LR analyses showed a reversed pattern: hummingbird‐syndrome species have significantly narrower, but not longer, corolla tubes compared with bee‐syndrome species (Table 1). In addition to finding that nectar traits are strongly associated with pollination syndrome, we found that nectar volume and nectary area exhibit a strong evolutionary association according to PGLS (*P* = 0.0127, model = PL, lambda = −0.255, slope = 0.775; Figs 2a, S2).

**Table 1:** Phylogenetically corrected association of quantitative floral traits with pollination syndrome using phylogenetic ANOVA and phylogenetic linear regression (LR)

Trait model	*P*‐values		Phylogenetic model for LR
	ANOVA	LR	
Pollinator ~ Corolla color	**0.001**	**4.1 × 10** ^**−10**^	OU
Pollinator ~ Nectary area	**0.015**	**0.027**	PL
Pollinator ~ Nectar volume	**0.01**	**0.021**	PL
Pollinator ~ Floral tube length	0.336	0.359	PL
Pollinator ~ Floral tube width	**0.003**	**0.002**	OU

Significant associations (*P* < 0.05) are in bold. Preferred model for phylogenetic residual error in LR analyses are listed as Pagel's lambda (PL) and Ornstein–Uhlenbeck (OU).

### Analysis of nectary cell size

We found that for the two species pairs sampled, (1) *P. neomexicanus*,* P. barbatus*, and (2) *P. amphorellae*,* P. kunthii*, glandular trichome cell size was significantly larger in nectaries of the hummingbird‐syndrome species than in closely related bee‐syndrome species (Fig. 2b).

### Trait correlations in the *P. amphorellae* × *P. kunthii* F_2_ population

**Figure 3:**
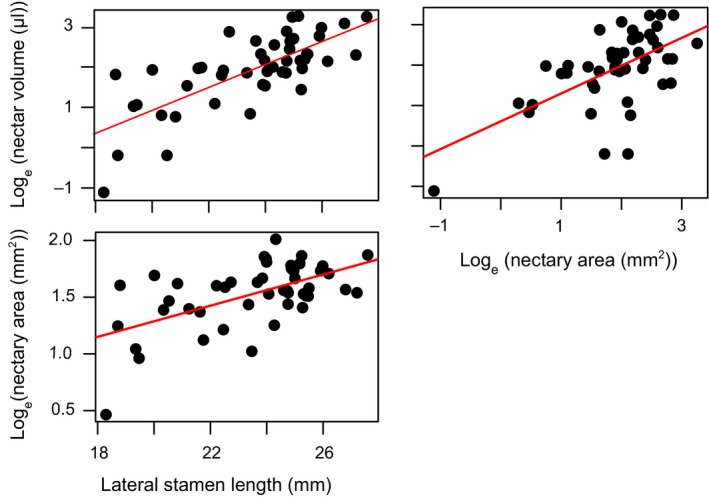
Trait correlations showing pairwise values and linear regression from 46 F_2_ individuals derived from a cross between bee‐adapted *Penstemon amphorellae* and hummingbird‐adapted *Penstemon kunthii*.

Within the *P. amphorellae* × *P. kunthii* F_2_ population, we found that nectar volume is positively correlated with nectary area (*P* = 7.24 × 10^−6^, *r* = 0.603; Fig. 3). In addition, we found that lateral stamen length is positively correlated with both nectar volume (*P* = 2.47 × 10^−9^, *r* = 0.742) and nectary area (*P* = 2.15 × 10^−5^, *r* = 0.577; Fig. 3).

## Discussion

### Nectar volume is a key correlate of pollination syndrome in *Penstemon*

Bright red to magenta flowers and large nectar rewards are considered the key traits required for a shift from insect to hummingbird pollination in the North American flora (Grant, 1992; Thomson *et al*., 2000; Wilson *et al*., 2006, 2007). Similar to Wilson *et al*. (2004), we find that flower color is strongly associated with pollination syndrome among North American *Penstemon*. Additionally, we find that nectar volume is significantly associated with pollination syndrome, corroborating qualitative results in Thomson *et al*. (2000). Together, these floral traits associated with pollination syndrome in *Penstemon* establish color and nectar reward as key attraction traits for evolutionary transitions to hummingbird adaptation.

Flower dimension traits are often ascribed to pollination syndrome. In *Penstemon*, flowers with longer, narrower corolla tubes, longer reproductive organs exserted from the corolla tube, and lack of a lower petal lobe landing platform are all considered adaptations to hummingbird pollination. Our results for corolla tube length and width association with pollination syndrome differ from those found in Wilson *et al*. (2004). Wilson *et al*. (2004) identified corolla tube length as more strongly associated with pollination syndrome than corolla tube width, whereas we found the opposite pattern. This difference is not surprising given that the overall size of *Penstemon* flowers varies widely, even within bee‐ and hummingbird‐syndrome classes (see Table S1). Here, we sampled 19 *Penstemon* species, of which only nine overlapped with the 49 species sampled in Wilson *et al*. (2004). Differences primarily reflect pollination mechanisms among the sampled bee‐adapted species. These differences in sampling across studies likely explain the different levels of association between dimension traits and pollination syndrome.

### Nectary area is a morphological correlate of nectar production in *Penstemon*

Elevated nectar production is a key trait that increases hummingbird visitation, a requirement for transitions to hummingbird pollination (Wilson & Jordan, 2009). Unlike the genetics of flower color evolution, which has been extensively studied with respect to shifts in pollination syndrome (Zufall & Rausher, 2004; Streisfeld & Rausher, 2009; Smith & Rausher, 2011; Wessinger & Rausher, 2014), little is known about the developmental or genetic basis for variation in nectar volume. Studies have begun to illuminate the genetic requirements for initiating nectary development (Bowman & Smyth, 1999; Morel *et al*., 2018; Min *et al*., 2019), but these provide little insight into the basis of interspecific variation in nectar production. Because of the many parallel transitions to hummingbird pollination, and the clear pattern we find here that nectar volume and nectary area are significantly associated with pollination syndrome, *Penstemon* is an excellent model to begin dissecting the developmental correlates of nectar volume.

We find increased nectar production is predictably associated with larger nectaries across six independent origins of hummingbird syndrome in *Penstemon* (Figs 2a, S2). This suggests that a developmental shift in nectary size may be an important mechanism for increased nectar reward in adaptive shifts to hummingbird pollination in *Penstemon*. Increased nectary size may be one part of a multifaceted transition to increased nectar production. In addition to nectary size, physiological changes to nectar‐secreting cells (Fahn, 1988; Ge *et al*., 2000) may also underlie the evolution of larger nectar volumes associated with hummingbird syndrome in *Penstemon*. For example, Ge *et al*. (2000) show that overexpression of the *SWEET* gene in petunia leads to increased phloem bundle accumulation in the nectary, which in turn could affect nectar volume.

### Cellular‐level processes contribute to interspecific variation in nectary area

Nectary area is 46.5% and 64.8% larger in hummingbird‐syndrome *P. kunthii* and *P. barbatus*, compared to their bee‐syndrome relatives *P. amphorellae* and *P. neomexicanus*, respectively. Glandular trichome cell size is respectively only 11.2% and 8.2% larger in the hummingbird species from these pairs. Therefore, larger nectary area associated with hummingbird syndrome is at least partially explained by larger individual glandular trichome cells forming the nectary. Because cell size does not appear to explain all the variation in nectary area, processes of cell proliferation are likely to additionally contribute to overall variation in nectary size. Although our nectary cell size results are from just two independent origins of hummingbird pollination in *Penstemon*, they do suggest that parallel developmental processes involving a combination of cell expansion and cell proliferation processes underlie interspecific variation in nectary area.

### Trait correlation of nectar volume with nectary area suggests a mechanistic relationship

We found that nectar volume is associated with nectary area across a sample of species. This suggests a mechanistic relationship between nectar volume and nectary size. Consistent with this hypothesis, nectar volume and nectary area are correlated in a segregating F_2_ population. The correlation is strikingly strong given that quantification of nectar volume is inherently noisy. Given our data supporting a mechanistic link between nectary area and nectar production, the response to selection for larger volumes of nectar, imposed by hummingbird pollinators, may involve (at least in part) the evolution of larger nectaries.

The genetic basis for differences in nectar volume and nectary size in *Penstemon* remains unknown. In future QTL studies aimed at identifying the genetic basis for adaptive differences in nectar volume, our data predict extensive QTL co‐localization of nectar volume with nectary area. We expect that candidate genes under such QTL for both nectar volume and nectary area should be associated with cell expansion or proliferation processes. Existing evidence points to a single QTL of large effect for variation in nectar volume between bee‐ and hummingbird‐adapted *Penstemon* species (Wessinger *et al*., 2014). Future characterization of this QTL provides an opportunity to test this hypothesis.

### Trait correlation of nectary area with stamen length may facilitate evolution of the hummingbird syndrome

*Penstemon* nectaries are intimately associated with lateral stamens, and stamen length is a key trait distinguishing pollination syndromes. Therefore, we tested whether nectary area is correlated with stamen length under the premise that an overall increase in stamen size could indirectly affect nectary area, or vice versa. In our *P. kunthii* × *P. amphorellae* F_2_ population, we found that nectary area and stamen length are positively correlated. Given that this positive correlation is in the direction of adaptation to hummingbird pollination, this correlation could facilitate multi‐trait adaptation, specifically if the correlation results from genetic linkage or pleiotropy.

Positive correlations between nectar traits and stamen length are not an intrinsic feature across *Penstemon*. Both nectary area and nectar volume are positively correlated with stamen length in the *P. kunthii* × *P. amphorellae* F_2_ mapping population studied here. However, in a previous cross between bee‐syndrome *P. neomexicanus* × hummingbird‐syndrome *P. barbatus*, F_2_s did not exhibit significant correlation between nectar volume and stamen length (Wessinger *et al*., 2014). Therefore, correlations may facilitate adaptation in some *Penstemon* lineages. In other lineages these traits may be uncoupled, allowing nectar and stamen length traits to respond independently to selection.

### Conclusions

Parallel genetic changes underlying multiple transitions to red‐flowered hummingbird‐adapted *Penstemon* have been well documented (Wessinger & Rausher, 2015; Wessinger & Hileman, 2016). Here, we demonstrate that nectar volume is a key trait associated with *Penstemon* pollination syndromes and that developmental processes associated with nectary size contribute to variation in nectar reward. We provide evidence for parallel processes of cell expansion and cell proliferation underlying repeated transitions toward increased nectar production in hummingbird‐syndrome *Penstemon*. Our analyses contribute to growing evidence that predictable and repeated developmental mechanisms are associated with hummingbird adaptation in *Penstemon*. Additionally, we suggest that positive correlation between nectar traits and stamen length may facilitate adaptive evolution in some, but not all, lineages of *Penstemon*.

## Author contributions

AMK, CAW and LCH co‐conceived the project, AMK and CAW collected data and analyzed data, and AMK, CAW and LCH co‐wrote the manuscript.

## Supporting information

Please note: Wiley Blackwell are not responsible for the content or functionality of any Supporting Information supplied by the authors. Any queries (other than missing material) should be directed to the *New Phytologist* Central Office.

**Fig. S1** *Penstemon* nectary morphology and microscopy.
**Fig. S2** Scatterplot depicting the association between nectar volume (μl) and nectary area (mm^2^), and species pairs contrasting in pollination syndrome highlighted.

**Table S1** *Penstemon* flower dimension and nectar volume measurements (mean and variance), and flower color designations, used for analyses of evolutionary trait associations.
