# Supplementary material for: Nectary size is a pollination syndrome trait in Penstemon
Source: New Phytol. 2019 Mar 26;223(1):377–84. doi: 10.1111/nph.15769 (PMC6593460; doi:10.1111/nph.15769)
Supplement: Supplementary file 1 — Fig. S1 Penstemon nectary morphology and microscopy. Fig. S2 Scatterplot depicting the association between nectar volume (μl) and nectary area (mm2), and species pairs contrasting in pollination syndrome highlighted. [file NPH-223-377-s001.pdf]

New Phytologist Supporting Information
Nectary size is a pollination syndrome trait in *Penstemon*
Amanda M. Katzer, Carolyn A. Wessinger, Lena C. Hileman
Acceptance date: 24 February 2019

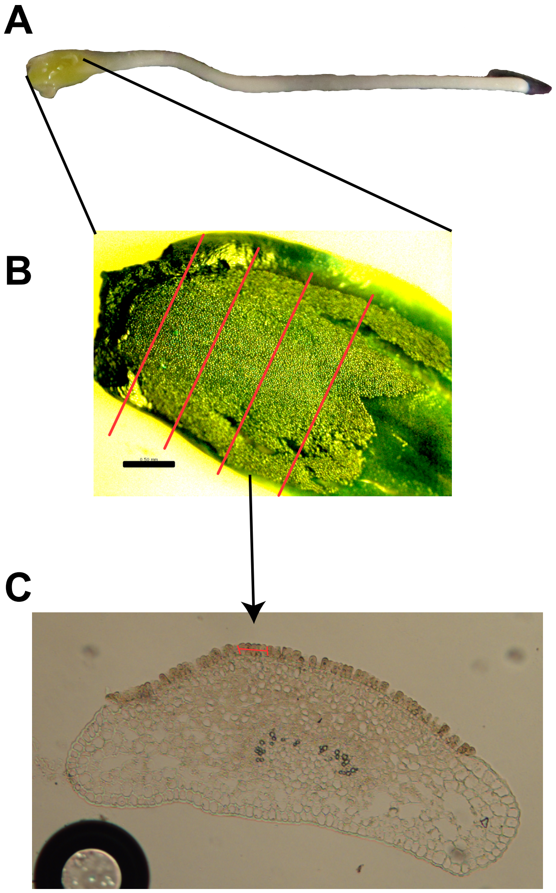

Supporting Information Figure 1. *Penstemon* nectaries showing position of nectary at base of lateral stamen filament (A); nectary with position (red lines) of sections from proximal to distal end used for trichome cell size quantification, scale bar = 0.5 mm (B); and magnified image of nectar-producing trichome cells on the surface of a nectary (C), red bracket shows how one 5-cell measurement across the nectary surface is made.

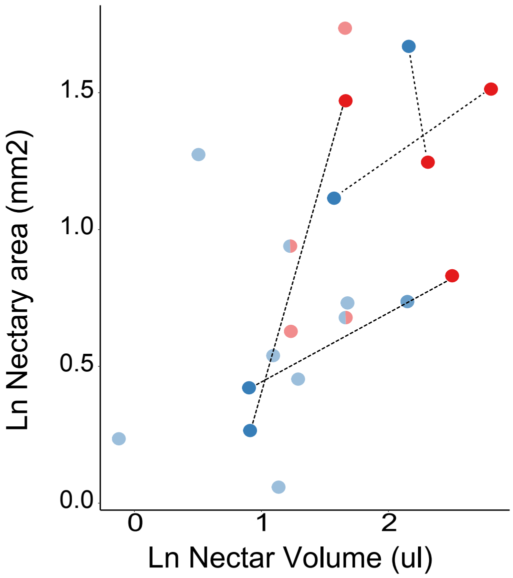

Supporting Information Figure 2. Scatterplot depicting the association between nectar volume (μl) and nectary area (mm2). Bee-adapted and hummingbird-adapted species are depicted in blue and red as in Figure 2a. Dotted lines connect species pairs that differ in syndrome from Figure 1.
